# Feasibility and preliminary findings of a bacterial diversity study in periodontitis: a pilot investigation from the Western Cape

**DOI:** 10.3389/froh.2025.1568393

**Published:** 2025-04-23

**Authors:** Salma Kabbashi, Yvonne Prince, Ndonwi Elvis Ngwa, Haly Holmes, Glenda Mary Davison, Saarah F. G. Davids, Manogari Chetty

**Affiliations:** ^1^ Department of Craniofacial Biology, Pathology, & Radiology, Faculty of Dentistry, University of the Western Cape, Cape Town, South Africa; ^2^ SAMRC/CPUT/Cardiometabolic Health Research Unit, Department of Biomedical Sciences, Faculty of Health & Wellness Sciences, Cape Peninsula University of Technology, Cape Town, South Africa; ^3^ Department of Oral Medicine & Periodontology, Faculty of Dentistry, University of the Western Cape, Cape Town, South Africa

**Keywords:** periodontitis, periodontal health, bacterial profile, 16S rRNA, *Fusobacterium*, diversity measures, South Africa

## Abstract

**Introduction:**

Periodontitis is a significant health challenge caused by a complex interaction between bacterial infection, host immune response, and environmental factors, leading to tooth loss, bone loss, and potential associations with major systemic diseases and conditions. While the determinants of periodontitis have been extensively investigated in other populations, such studies are lacking in South Africa, which represents a high-risk population. Therefore, this study was conducted to characterize the subgingival bacterial biodiversity in the periodontal pockets of patients with periodontitis in a Western Cape population.

**Materials & methods:**

Pooled subgingival plaque samples were collected from the deepest pocket/crevices of five periodontitis cases and five controls using sterile paper points. Illumina MiSeq paired-end sequencing and QIIME2 software were employed for sequence filtration and analysis. Several alpha and beta-diversity metrics assessed biodiversity within-sample and population structure between different microbiota datasets, respectively. Statistical significance for alpha diversity was tested using the Kruskal–Wallis H test (*p* < 0.05), and beta diversity differences were evaluated using PERMANOVA. Data visualization, including beta diversity plots, was conducted with the Phyloseq package in R.

**Results:**

Beta-diversity measures revealed significant differences between periodontitis cases and controls (*p*-value = 0.04), whereas alpha-diversity was higher in cases, though without statistical significance (*p*-value ≥ 0.05). Cases group showed high relative abundance of *Fusobacterium* (16%), *Porphyromonas* (10%), and *Treponema* (9%), while the periodontally healthy controls were dominated by *Streptococcus* (20%), *Fusobacterium* (15%), and *Veillonella* (10%), with *g_Streptococcus* showing a significant difference (*p*-value = 0.008). Differential abundance analysis revealed distinct bacterial genera enriched in cases (*Bulleidia*, *Peptoanaerobacter*, *Phocaeiola*, *W5053*) and controls (*Abiotrophia*, *Haemophilus*, *Lautropia*, *Rothia*, *Streptococcus*). Sample-specific variations included higher levels of *Porphyromonas* (15%) in grade B and *Fusobacterium* (20%) in grade C.

**Conclusion:**

This exploratory study highlights distinct bacterial communities associated with periodontitis in a South African population. The findings emphasize the need for larger, population-based cohorts to validate these results and lay a foundation for future research into region-specific microbial profiles and their implications for personalized treatment strategies.

## 1 Introduction

According to the WHO's Global Oral Health Status Report (2022), 19% of the global population aged 15 years and older is affected by severe periodontitis, with the highest prevalence in Africa (23%) (1). Periodontitis is a multifactorial disease triggered by dysbiosis in dental biofilm that promotes inflammation with both protective and destructive effects on periodontal tissues (2). This dysbiosis negatively impacts oral and general health, reducing the quality of life (3) and contributing to a global healthcare burden and social inequality (4).

Advances in culture-independent molecular techniques have revealed distinct bacterial communities in periodontal health and disease (5). Despite limitations such as short sequencing reads, limited species-level resolution for certain bacterial genera (6), lack of standardization in selecting hypervariable regions for sequencing (7), and an inability to provide functional insights (8), 16S rRNA sequencing remains the gold standard for bacterial profiling (9). These techniques highlight the diagnostic and prognostic potential of microbiome biodiversity in periodontitis, where changes in microbial composition signal early disease, and biodiversity loss is linked to disease progression (10). Restoring microbial diversity could be a key therapeutic goal in periodontitis, promoting a balanced oral microbiome essential for achieving and maintaining periodontal stability (11).

While the current classification system for periodontitis incorporates pathophysiological and host immune factors that justified the consolidation of periodontitis into a unified category (12), it does not include microbial criteria to differentiate between stages and grades. This limitation arises primarily from insufficient evidence-based data on microbiological diagnostics in periodontitis (13).

Emerging evidence suggests that the subgingival microbial composition in periodontitis patients varies significantly based on population, demographic factors, and environmental conditions (14, 15). These findings highlight the potential relevance of microbial profiling in enhancing and refining periodontal classification systems.

However, much of our understanding of periodontitis microbiota is derived from studies conducted in non-African populations (16). Recent insights highlight the need for population-specific investigations. For instance, *Aggregatibacter actinomycetemcomitans*, strongly linked to Grade C Molar-Incisor periodontitis, appears to behighly prevalent among Africans, compared to other ethnic groups" (17–20).

Therefore, investigating periodontitis determinants in high-risk, diverse populations, such as South Africans (SAs), is imperative for developing a successful precision dentistry health system. Recent work established the microbial profile of the SA population who smoke (21). However, no study has been conducted to establish the baseline bacterial profile of SAs with periodontitis compared to those with a healthy periodontium.

Given these considerations, the primary objective of this study was to characterize the subgingival bacterial communities within periodontal pockets of periodontitis patients from the Western Cape, SA, and to assess whether microbial composition differs across the various grades of periodontitis. This pilot study aims to lay the foundation for future, more comprehensive research by evaluating both the feasibility and potential benefits of the proposed study within a diverse population.

## 2 Material and methods

### 2.1 Study population

Ethical approval was obtained from the Biomedical Research Ethics Committee (BMREC) of the University of the Western Cape (UWC), reference number BM20/10/9. Twenty-three consecutive South African participants, aged 18 years or older and with at least 10 teeth, were recruited from the Oral Medicine and Periodontology Department at Tygerberg Oral Health Centre between August 2021 and October 2022. Participants were excluded if they had any systemic disease, were current smokers, pregnant or lactating, edentulous, had used antiseptics or anti-inflammatory drugs for more than one week, antibiotics, antimicrobials, undergone any periodontal management in the past three months. Therefore, a final sample size of 23 patients was included in this study.

All potential participants underwent Basic Periodontal Examination (BPE) to assess their periodontal status. Eligible participants then received a comprehensive periodontal examination for detailed clinical data collection. Personal and demographic information, including age, gender, and self-reported ethnicity, were also collected.

Panoramic radiographs were taken, and a calibrated periodontist with high intra-reliability (kappa > 0.81) conducted clinical examinations, documenting periodontal parameters such as full mouth plaque score (FMPS), full mouth bleeding score (FMBS), probable pocket depth (PPD), and clinical attachment level (CAL). Participants were categorized into periodontitis cases and periodontally healthy controls based on the case definitions outlined in the 2018 classification scheme of periodontal diseases (13, 22). Following this, a complete diagnosis of periodontitis cases was established using the multidimensional staging and grading systems, in which disease severity was categorized into four stages (I–IV), ranging from mild clinical attachment loss, Stage I, to severe periodontitis with extensive bone and tooth loss, Stage IV. Staging was determined based on CAL, radiographic bone loss, disease complexity, and its extent and distribution; localized or generalized. Progression was further graded as A (slow progression), B (moderate progression), or C (rapid progression). Grading was based on CAL and bone loss in relation to age, case phenotype reflected through FMPS, and the presence of grade modifiers such as diabetes mellitus (DM) and smoking (13, 22).

### 2.2 Subgingival sampling

After the removal of the supra-gingival biofilm, the teeth targeted for sampling were isolated with cotton rolls and dried. Sub-gingival dental biofilm samples were acquired from the meso-buccal surface of teeth with the deepest pathological pocket (or cervices in the healthy group) of each quadrant by gently introducing a sterile paper point #35 (23–30).

If a deeper pathological pocket (or cervices in the healthy group) was identified on another surface, it was used for sample collection. Paper points were immediately transferred from the participant's mouth and pooled into a sterile 2 ml Eppendorf tube containing 500 μl of phosphate-buffered saline (PBS), then immediately placed on ice. As per the recommendation of the best storage temperature for microbiome material, samples were frozen at −80°C at IMBM until DNA extraction was conducted.

### 2.3 DNA extraction and sequencing

DNA extraction from the dental biofilm samples was performed using the PureLink™ Microbiome DNA Purification Kit (#A29790; Thermo Fisher Scientific, Waltham, Massachusetts, USA), following the manufacturer's recommendations under sterilized conditions. The extracted DNA quality and concentration were evaluated using the NanoDrop® ND 1,000 Spectrophotometer and the Qubit® 2.0 fluorometer (Invitrogen, Thermo Fisher Scientific, Waltham, Massachusetts, USA), respectively. Samples with DNA quality metrics meeting the Illumina 16S metagenomics workflow requirements for optimal outcomes (≥20 μl of 10 ng/μl, A260/280: 1.8–2.0, A260/230: 1.5–2.2) were selected for downstream analysis. While the majority of the samples met the criteria, only the top ten with the highest quality and quantity were included in downstream analysis due to funding limitations.

For PCR amplification of the hypervariable V3-V4 regions of the16S ribosomal RNA gene (16S rRNA), the following primer sequences were used:

• **Forward primer** (341F primer + Illumina overhang adapter underlined): TCGTCGGCAGCGTCAGATGTGTATAAGAGACAGCCTACGGGNGGCWGCA
• **Reverse primer** (805R primer + Illumina overhang adapter underlined): GTCTCGTGGGCTCGGAGATGTGTATAAGAGACAGGACTACHVGGGTATCTAATCC

Following this, the Nextera XT v2 Indexes were used for amplicon barcoding, which were then multiplexed, spiked with 10% of a 6 pM PhiX, and sequenced using the MiSeq Reagent Kit v2 (500 cycles) on the Illumina MiSeq platform (#15044223, Illumina, San Diego, CA, USA) at the Centre for Proteomic and Genomic Research.

### 2.4 Data analysis

Raw sequencing reads were quality filtered and trimmed, retaining only a Q-score > 20 and overlapping regions were allowed up to two of the ambiguous bases. Analysis of raw sequence data was mainly performed using Quantitative Insights into Microbial Ecology 2 (QIIME2) 2022.2 (https://qiime2.org/, RRID:SCR_021258), with DADA2 plugin to ccorrect sequencing errors and cluster the sequences into amplicon sequence variants (ASVs). Each ASV was aligned and classified at the genus level using the Greengenes database, V.2024.09 (https://qiime2.org/, RRID:SCR_002830).

Differential abundance testing was conducted by the Wald test with Benjamin-Hochberg adjustment for false discovery (31) using an adjusted *p*-value < 0.05, as implemented in the DESeq2-package in R. Features appearing in <10% of the samples and a relative abundance of <5% were filtered out.

Further statistical analysis and plot generation were conducted using R package, V4.2 (http://www.r-project.org/, RRID:SCR_001905).

Alpha-diversity was assessed using various metrics, including Observed richness, Chao1, Abundance Coverage Estimator (ACE), Simpson and Shannon indices, to estimate richness, abundance, and diversity within the sample (32). The Kruskal–Wallis H test was applied to all alpha-diversity metrics to determine statistical significance, with a threshold set at *p*-value < 0.05.

Beta-diversity was evaluated using the Bray-Curtis, Jaccard, Weighted UniFrac, and Unweighted UniFrac distance metrics on the rarefied datasets to assess differences in microbial community structure between comparison groups. The Phyloseq package in R was used to visualize these distances through principal coordinate analysis (PCoA) plots. Distance-based permutation multivariate analysis of variance (PERMANOVA) was conducted using Adonis 2 from the vegan R package (33). This was followed by a test for homogeneity.

## 3 Results

### 3.1 Demographics and clinical parameters

The demographics and clinical parameters are detailed in Table 1. The majority of the participants in the study were females, with only one male in the control group. Within the control group, three participants self-identified as SA Coloured (SAC), while the case group had an equal distribution of Caucasian and African participants, with only one participant from the SAC ethnic group. Among the periodontitis cases examined, Stage IV was the most prevalent, observed in three out of five cases, while Stage III accounted for the remaining two. For grading, the cases were classified as either Grade B or C, with Grade C indicating the highest severity, also found in three out of five cases. Additionally, all periodontitis cases exhibited a generalized distribution, as detailed in Table 1. *P*-values for the participant's quantitative and qualitative variables were not included as this was a preliminary descriptive study with limited sample size.

**Table 1:** Participants demographic and periodontal parameters.

Participants	Age years	Sex	Race	Present teeth	FMPS %	FMBS %	PPD mm	CAL mm	Diagnosis	Grade
Controls										
1	59	M	SAC	21	12	0	2.4	3.1	Periodontal Health	-
2	20	F	Asian/Indian	28	14	2	2.4	2.4	Periodontal Health	-
3	20	F	SAC	32	10	5	2.4	3.1	Periodontal Health	-
4	65	F	Caucasian	22	0	11	3.0	3.1	Periodontal Health	-
5	32	F	SAC	29	7	10	2.6	2.5	Periodontal Health	-
Cases										
1	40	F	SAC	25	31	26	4.2	4.5	Generalized Periodontitis stage IV grade C	C
2	41	F	Caucasian	25	62	47	4.2	4.2	Generalized Periodontitis stage IV grade B	B
3	54	F	African	21	68	33	3.6	3.7	Generalized Periodontitis stage IV grade C	C
4	36	F	African	25	29	86	3.5	3.6	Generalized Periodontitis stage III grade B	B
5	65	F	Caucasian	25	17	15	3.4	4.5	Generalized Periodontitis stage III grade C	C

M, male; F, female; FMPS, full mouth plaque score; FMBS, full mouth bleeding index; PPD, probable plaque depth; CAL, clinical attachment loss; mm, millimetre.

The observed distribution of the continuous variables between cases and controls is tabulated in Table 2. Based on the full-mouth clinical examination, periodontitis cases exhibited higher FMBS, FMPS, PPD, and CAL values, and fewer present teeth, when compared to the periodontally healthy controls. The differences between the median estimates all seem minimal. The control subjects seemed to be about 8 years younger on average but with larger variation between them (SD ≈ 21.49).

**Table 2:** The distribution of the continuous variables between periodontitis cases and controls.

Variable	Cases (Median, 25th–75th Percentile)	Control (Median, 25th–75th Percentile)
Age (years)	41.0 (38.0–59.5)	33.0 (21.5–62.0)
Teeth Present	25.0 (25.0–25.0)	28.0 (22.0–29.0)
FMPS (%)	31.0 (29.0–62.0)	10.0 (7.0–12.0)
FMBS (%)	33.0 (26.0–47.0)	5.0 (2.0–10.0)
PPD (mm)	3.6 (3.4–4.2)	2.4 (2.4–2.6)
CAL (mm)	4.2 (3.7–4.5)	3.0 (5.5–3.0)

FMPS, full mouth plaque score; FMBS, full mouth bleeding index; PPD, probable plaque depth; CAL, clinical attachment loss; mm, millimetre.

### 3.2 Sequence data

A total of 3.2 million V3–V4 16S rDNA paired-end reads were generated from the 10 pooled samples. After filtering, 2.7 million reads were left. The average length of the filtered reads was 599 bp. A total of 1,482 features were resolved, with a total frequency of 680,617 after quality filtering.

### 3.3 Subgingival bacterial communities

#### 3.3.1 Bacterial compositions

**Figure 1:**
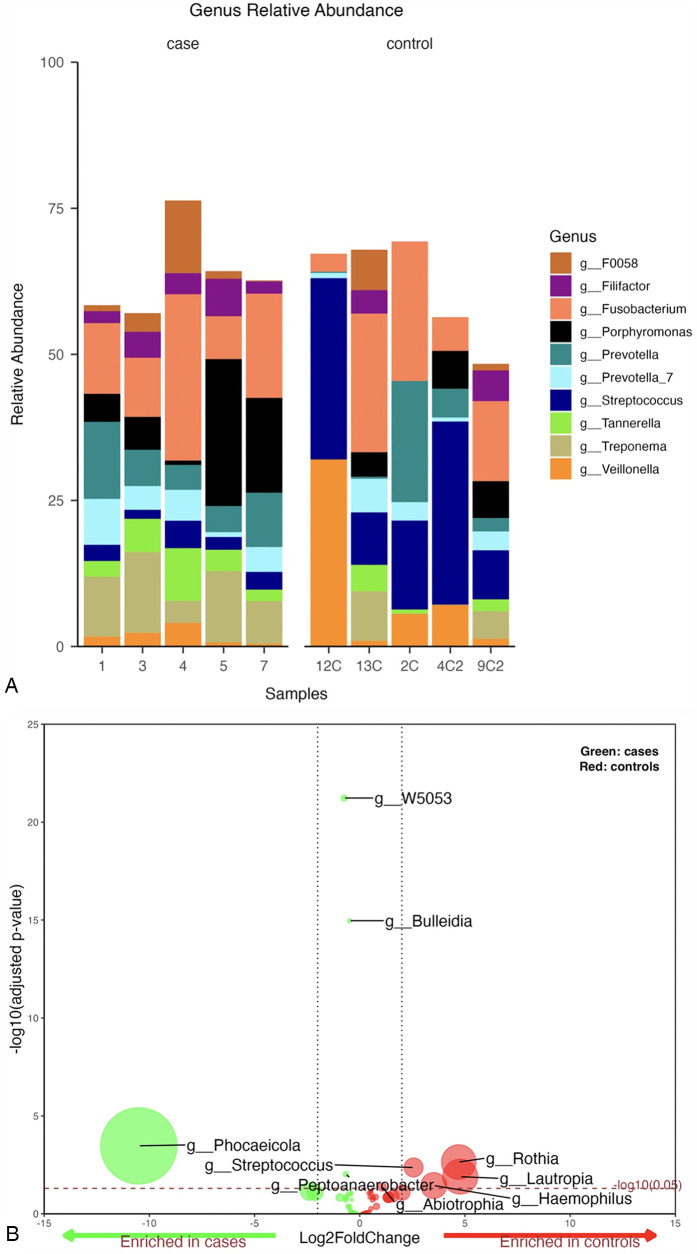
**(A)** Mean relative abundance of the top 10 genera generated from periodontitis cases vs. control samples, **(B)** Volcano plot depicting differentially abundant taxa as determined by DESeq2 in cases vs. controls at genus level. Significantly more discriminatory taxa appear above the threshold (red dotted line, FDR = 1). The relative abundance of taxa is indicated by circle size.

The top 10 genera with the highest relative abundance in the periodontitis cases group were *Fusobacterium* (16%), *Porphyromonas* (10%), *Treponema* (9%), *Prevotella* (8%), *Prevotella_7* (5%), *Tannerella* (5%), *Filifactor* (4%), *F0058* (4%), *Streptococcus* (3%), and *Veillonella* (2%) (Figure 1A). In contrast, the control group was dominated by *Streptococcus*, accounting for 20% of all bacteria at the genus level. This was followed by *Fusobacterium* (15%), *Veillonella* (10%), *Prevotella* (7%), *Porphyromonas* (3%), *Treponema* (3%), *Prevotella_7* (3%), *Tannerella* (2%), *Filifactor* (2%), and *F0058* (2%) (Figure 1A).

A pairwise differential abundance analysis at the genus level showed that four genera, *Bulleidia, Peptoanaerobacter, Phocaeiola,* and *W5053,* were enriched in the cases group samples, while five genera, *Abiotrophia, Haemophilus, Lautropia, Rothia*, and *Streptococcus*, were enriched in the controls (Figure 1B).

A statistically significant difference was only noted for the *g_Streptococcus* when comparing the top 10 genera between periodontitis cases and periodontally healthy controls at genus level (*p*-value = 0.008) (Supplementary Figure S1).

**Figure 2:**
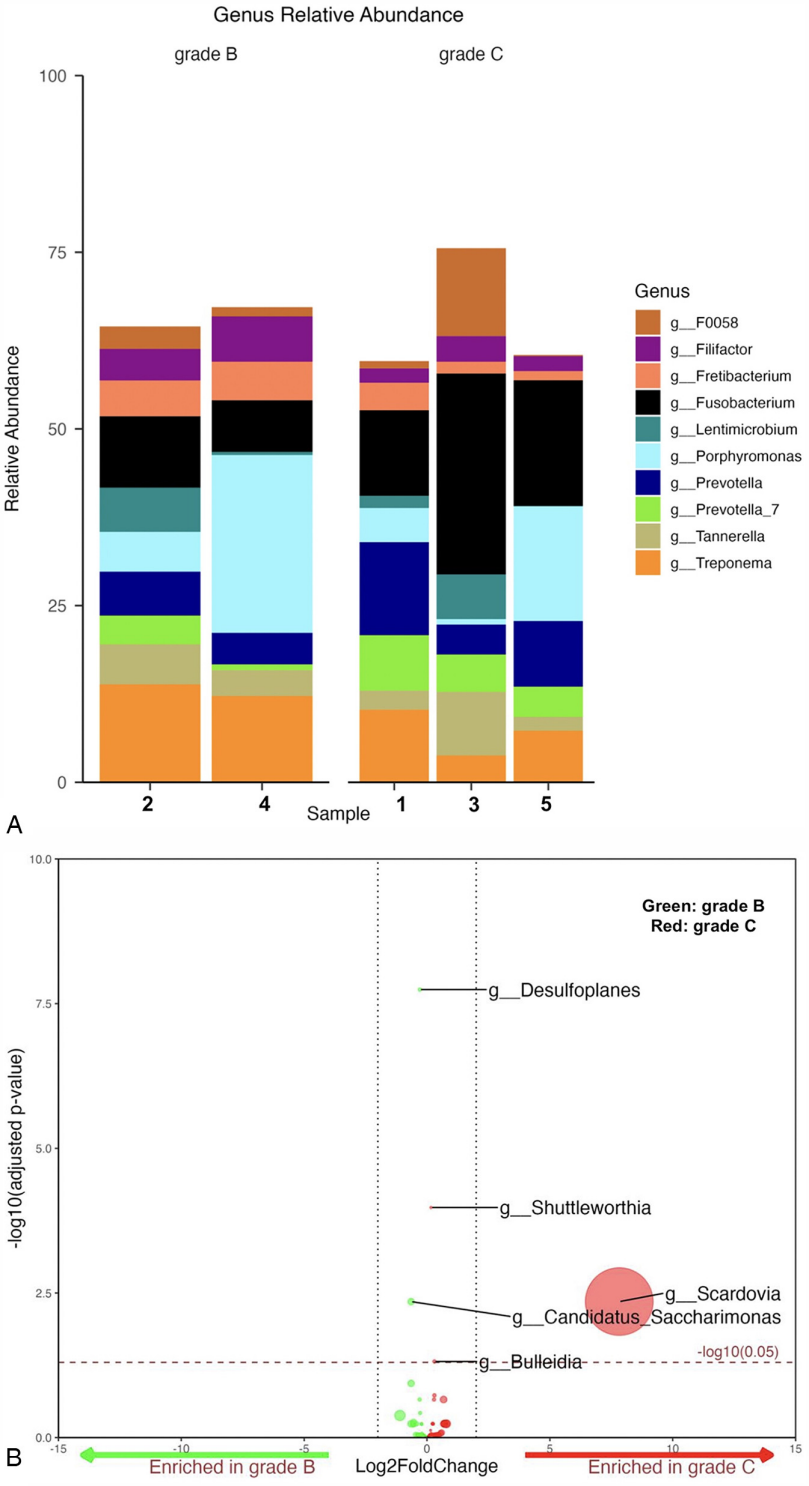
**(A)** Mean relative abundance of the top 10 genera generated from grade B vs. C samples. **(B)** Volcano plot depicting differentially abundance taxa as determined by DESeq2 in grade B vs. C at genus level. Significantly more discriminatory taxa appear above the threshold (red dotted line, FDR = 1). Relative abundance of taxa is indicated by circle size.

Among periodontitis samples, *Fusobacterium* was the most abundant genus (20%) in grade C, while *Porphyromonas* (15%) and *Treponema* (13%) were more dominant in grade B. In grade B, sample 2 showed a relatively equal abundance of all detected genera, with slightly higher levels of *Treponema* and *Fusobacterium*. Sample 4 was unique in having a distinctively higher abundance of *Porphyromonas* among all samples. All grade C samples were dominated by *Fusobacterium*. However, sample 3 had higher abundance of *Tannerella* and *F0058*, followed by *Lentimicrobium*. Samples 1 and 5 had high levels of *Prevotella* and *Treponema*, with sample 5 particularly rich in *Porphyromonas* and depleted in *Lentimicrobium* (Figure 2A).

A total of five taxa were found to be significantly differentially abundant (Figure 2B). Of the five, *Desulfoplanes* and *Candidatus Saccharimonas* were enriched in grade B cases, while *Shuttleworthia, Bulleidia*, and *Scardovia* were enriched in the grade C cases. No other statistically significant differences were identified among all genera between both grades (*p*-value ≥ 0.059) (Supplementary Figure S2).

#### 3.3.2 Bacterial diversity

Despite observing higher median alpha-diversity in the periodontitis samples compared to the controls (Supplementary Figure S3), there was no statistically significant difference (all *p*-values > 0.05) in the alpha-diversity metrics used. Borderline significant *p*-values for Shannon and ACE suggest potential subtle differences (Dunn test, Table 3). Values of multiple alpha diversity and richness indices conducted for cases and controls group samples are presented in Supplementary Table S4.

**Table 3:** *P*-values for alpha-diversity indices in periodontitis cases and controls.

Alpha_metric	Kruskal-*p*-value	Dunn-*p*-value
Observed	0.1172	0.0586
Simpson	0.6015	0.3008
Chao1	0.1172	0.0586
Shannon	0.0758	0.030
ACE	0.0758	0.0379

ACE, abundance coverage estimator.

**Figure 3:**
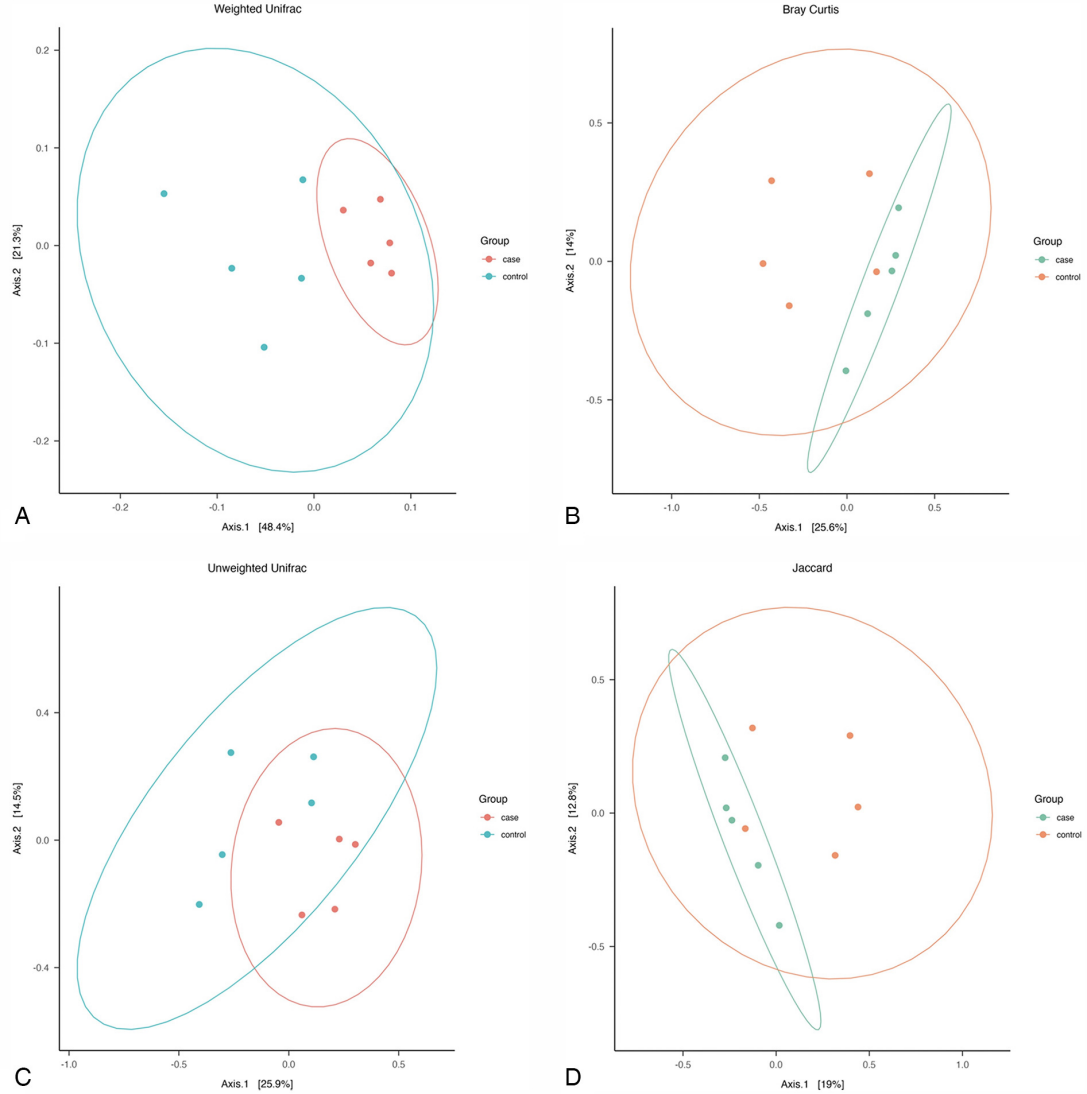
Pcoa plot of: **(A)** weighted unifarc distance, **(B)** bray curtiz dissimilarity distances, **(C)** unweighted unifrac distances, and **(D)** jaccard distances, across all samples.

The phylogeny-based Weighted-Unifrac distance matrix explained the highest amount of variance (69.7%) compared to Bray Curtis, Unweighted Unifrac, and Jaccard measures, which explained 39.6%, 40.4%, and 31.8% of the variance, respectively. The PCoA plot based on Weighted-Unifrac distance matrix showed that all cases samples clustered together, while the controls samples appeared more dispersed (Figure 3). PERMANOVA tests revealed significant differences in the average community composition between cases and controls (*p*-value = 0.04) but with a non-significant homogeneity condition test result (*p*-value = 0.16) (Table 4).

**Table 4:** PERMANOVA and PERMANOVA homogeneity tests for beta diversity conducted between cases and control groups.

Permonova test					
Df	Sums of Sqs	Mean Sqs	F.Model	R2	Pr.F.
1	0.53	0.53	1.60	0.17	0.04*
8	2.64	0.33	NA	0.83	NA
9	3.17	NA	NA	1.00	NA
Homogeneity test					
Df	Sum.Sq	Mean.Sq	F.value	Pr.F.	
1	0.01	0.01	2.44	0.16	
8	0.02	0.00	NA	NA	

Df, dgree of freedom; SumsOfSqs, sum of squares; MeanSqs, mean sum of square; F.Model, F-statistic model; R2, coefficient of determination; Pr.F., *P*-value of F statistic.

* Statistical significance.

## 4 Discussion

In this study, the subgingival microbiota in patients with periodontitis within a SA cohort, was characterised. The analysis demonstrated that, although the progression of periodontitis did not exhibit notable variations in bacterial diversity as assessed by alpha-diversity metrics, significant differences in community structure were identified using multiple beta-diversity indices.

The Weighted UniFrac distance analysis confirmed distinct clustering within the periodontitis group, whereas the control group exhibited greater dispersion, suggesting higher heterogeneity in microbiota composition and structure among healthy individuals.

These findings are consistent with those of Shi et al. (34), who utilized the Unweighted UniFrac distance metric to evaluate microbial variation in 25 periodontally healthy Chinese individuals, and Lenartová et al. (35), who employed the Bray-Curtis index to investigate microbial composition in 151 Czech participants across different clinical statuses and age groups. Both studies identified individualized subgingival plaque structures and attributed the dissimilarity observed in healthy biofilms to the presence of shallow gingival sulci, permitting environmental factors to influence microbial composition.

The “microbial succession” model dictates that several perio-pathogens initially invade the healthy microbiota, developing a diverse community of both health and disease-associated microbiota. As disease progresses, the transitional microbial community is then replaced by predominantly disease-associated organisms, resulting in the development of a more homogenous microbiota (10).

Findings from this study are consistent with studies conducted in Chilean, English, Chinese, and French populations (14, 36–38). They also partially align with the results of Nibali et al. (39), who reported significant differences in alpha and beta diversity across ethnic groups in England. However, our findings contrast with Schulz et al.'s (40), who observed no significant diversity differences in patients with aggressive periodontitis of Caucasian descent from Central Germany.

Several taxa exhibited comparable abundances between cases and controls, making it challenging to categorize them into distinct health status groups across studies (14, 35, 41). This challenge may stem from the potential limitations in accurately assigning health status at the microbial level, as such classifications are often based on clinical criteria. This issue was evident in the present investigation, where the relative abundances of certain genera showed minimal variation between cases with periodontitis and controls, including *Fusobacterium* (16% vs. 15%), *Prevotella* (8% vs. 7%), *Prevotella_7* (5% vs. 3%), *Tannerella* (5% vs. 2%), *F0058* (4% vs. 2%), and *Filifactor* (4% vs. 2%). In contrast, notable differences were observed in *Veillonella* (2% vs. 10%), *Streptococcus* (3% vs. 20%), *Porphyromonas* (10% vs. 3%), and *Treponema* (9% vs. 3%), with *Streptococcus* being significantly more abundant in controls (*p* = 0.008).

This investigation found *Fusobacterium, Porphyromonas, Treponema, Prevotella, Provetella_7, Tannerella, Filifactor,* and *F0058* genera to be closely associated with periodontitis, while *Streptococcus* and *Veillonella*, typically linked to healthy periodontium, showed decreased abundance.

These findings align with previous research and reaffirm the typical taxa associated with periodontitis, as demonstrated by decades of studies across diverse populations (37, 42–45). Notably, *Fusobacterium* emerged as the most dominant genus in this study, surpassing *Porphyromonas*, which remained highly abundant. Similar results were reported in Chilean and American populations using 454-pyrosequencing (14, 42), and in a Chinese cohort where *Fusobacterium* was detected in all chronic periodontitis (CP) patients and in over 86% of healthy controls using real-time PCR (46). In contrast, Kumar et al. (47) found *Fusobacterium* to be rare in healthy controls and nearly absent in individuals with shallow or deep pockets. Although *Fusobacterium* is generally elevated in periodontitis patients who smoke (21), the participants in this study were non-smokers.

One plausible explanation for this study finding is that, although *Porphyromonas gingivalis* is widely recognized as the key pathogen for periodontitis development, it is significantly less invasive to host cells than *Fusobacterium* species, as noted by De Andrade et al. (48). *Fusobacterium* demonstrates a stronger adhesion capacity to human gingival cells relative to *P. gingivalis*, thereby facilitating superior colonization (49). Furthermore, Zhang et al. (50) showed that co-infection with *Fusobacterium nucleatum* enhances the invasive potential of *P. gingivalis* in oral epithelial cells. This “promoter” capability of *F. nucleatum* underscores its critical role in mixed infections. Additionally, the high prevalence of *Fusobacterium* has been linked to various diseases, including colorectal and gastric cancers, across different populations (51–53). Consequently, the abundance of *Fusobacterium* in the SA population with periodontitis may suggest a population-specific predisposition.

In this study, *Filifactor* ranked among the top 10 genera in both periodontitis cases and controls. *Filifactor alocis* displays virulence traits that facilitate its persistence in periodontal pockets, thereby contributing to disease progression (54). The abundance of *Filifactor* varies across populations with CP, with prevalence rates of 30% in Swedish population (55), 66.7% in German population (56), and 83% in deep pockets and 36% in shallow pockets among Koreans (57).

The *F0058* genus, constituting 4% of cases and 2% of controls, was previously reported as abundant in Down syndrome patients with periodontitis (58), and in the salivary microbiome of Papillon–Lefèvre syndrome patients with neutrophil impairment (59). Its role in periodontitis warrants further investigation.

In the healthy group, *Streptococcus* and *Veillonella* were the predominant genera, consistent with findings from previous studies (35, 60–62). *Streptococcus*, a primary colonizer on clean tooth surfaces, plays a key role in promoting microbial adhesion (63), while *Veillonella*, considered a biomarker of health, consumes lactic acid produced by *Streptococcus mutans* and is associated with successful periodontal therapy (64). P. Zhou et al. (65) proposed that *Veillonella* functions as an “accessory pathogen”, facilitating the growth of pathogenic species within dysbiotic biofilms.

*Fusobacterium* was detected in 16% of healthy controls. While commonly present in a healthy periodontium, studies by Han (49) and Șurlin et al. (53) have also implicated *Fusobacterium* in the transition from gingivitis to periodontitis and its progression to more severe stages. *Fusobacterium* serves as a bridge between gram-positive symbionts and gram-negative pathogens in dysbiosis (66). Additionally, it has been shown to inhibit neutrophil proteases, thereby controlling tissue damage and providing defense against *P. gingivalis* (67).

*Prevotella* was found in comparable abundance (8% vs. 7%), consistent with prior studies (37, 42–45).Notably, sample 4 from the healthy controls exhibited a unique microbial profile, predominantly composed of *Veillonella* and *Streptococcus*, with periodontal pathogens such as *Treponema*, *Tannerella*, *Filifactor*, and *F0058* either absent or present in insufficient abundance. This atypical composition may be attributed to factors such as diet or the patient's advanced age, both of which can reduce microbial diversity (68). Further studies with larger sample sizes are needed.

*Peptoanaerobacter* showed higher differential abundance in the periodontitis group, highlighting its recently recognized role in exacerbating inflammation and its prevalence in periodontitis biofilms (69). Less common genera, including *Phocaeiola* (gut-associated), *W5053* (Firmicutes phylum), and *Bulleidia* (fecal origin), were also observed. While these genera have occasionally been detected in subgingival plaque of periodontitis patients (63–65, 70–72), their roles remain uncertain. However, these findings support the principle of multiple organisms contributing to biofilm-related disease, as previously described (73).

The control group showed a high differential abundance of genera linked to periodontal health including *Streptococcus*, *Rothia*, and *Abiotrophia* (35, 47, 74). *Haemophilus*, associated with *Streptococcus* in healthy periodontium (61, 75), and *Lautropia*, more common in younger individuals (35), were also observed.

To assess the risk of periodontitis progression based on microbial composition, bacterial abundance was compared between grade B and grade C cases. In moderate-risk grade B cases, *Porphyromonas* (15%) and *Treponema* (13%) were the dominant genera, consistent with their roles as keystone pathogens of the red complex, which induce dysbiosis and exhibit synergistic virulence in subgingival plaque (76, 77). In contrast, in grade C, associated with rapid disease progression, the abundance of the previously mentioned genera decreased (7% for each), while *Fusobacterium* (20%) and *Prevotella* (9%) became more dominant. The abundance of *Prevotella* was similar in both grades, as species such as *P. intermedia* and *P. nigrescens* from the orange complex are strongly linked to periodontitis (35, 44, 78) and are found in high abundance in both moderate and severe stages of periodontitis (79).

This study found that as periodontitis progresses from grade B to C, *Fusobacterium* abundance significantly increases. Unlike *P. gingivalis, Fusobacterium* potentiates inflammation by stimulating IL-8 and other virulence factors (49), explaining its persistence. However, the findings of this study contrast with those of a monozygotic twin study, where *Fusobacterium* was dominant in the grade B twin, while *Treponema* and *Fretibacterium* were more abundant in the grade C twin (80). The differences in severity between the twins, as well as the discrepancy with the present study's findings, may be attributed to the small sample size and non-shared environmental factors, such as long-term smoking in the grade C twin and corticosteroid use in the grade B twin (80).Additionally, a study in Brazil found higher *P. gingivalis* prevalence in grade C, suggesting ethnic and geographic variability (81).

*Scardovia* was differentially abundant in grade C patients, aligning with an animal study (82), though a pilot study found *Scardovia wiggsiae*, more common in periodontal health (83). This discrepancy may result from differences in sequencing techniques used (84). *Candidatus saccharimonas* typically present in low abundance, was differentially abundant in grade B samples, with elevated levels linked to severe periodontitis (37, 61, 85), though its role remains unclear (86). *Shuttleworthia* and *Bulleidia*, enriched in grade C, are associated with periodontitis progression (34, 87).

This pilot study had several limitations, primarily due to financial and resources constraints, which restricted the sample size to ten participants. These participants were selected based solely on the highest DNA purity and quantity measures, regardless of demographic matching. This introduced statistical limitations (e.g.,: the absence of *p*-values for quantitative and qualitative variables), and prevented comparisons based on gender or ethnicity. While previous research (88) was unable to identify sex-specific oral bacteria using 16S rRNA, the small sample size limits generalization. Age may impact oral microbial composition by reducing bacterial diversity (89). Future studies should use age-matched participants, a larger sample, and explore bacterial profiles across ethnic groups in SA to enhance generalizability and clinical reference. Finally, the recent development of periodontitis staging and grading systems limited the ability to draw definitive comparisons and conclusions.

However, this study has strengths in supporting the polymicrobial synergy and dysbiosis model by identifying shared bacteria like *Fusobacterium* between both groups, as well as underappreciated bacteria such as *F0058* and *Peptoanaerobacter*, which mediate inflammation. Additionally, the study cohort, consisting of SA with diverse ethnic admixtures, may further influence microbial composition due to genetic and environmental factors (14), highlighting population-specific patterns. By analyzing bacterial abundance across periodontitis grades (B and C), the study provides insights into the microbiota's role in disease progression.

## 5 Conclusion

This pilot study highlights the complexity of the oral microbiome in periodontitis, providing preliminary data that may inform future analyses extending beyond 16S sequencing. These analyses could further explore the role of *Fusobacterium* and the overall composition of the microbiota, with particular focus on population-specific patterns.

## Data Availability

The datasets presented in this study can be found in online repositories. The names of the repository/repositories and accession number(s) can be found below: https://www.ncbi.nlm.nih.gov/, PRJNA1110749.
