# Correction: Feasibility and preliminary findings of a bacterial diversity study in periodontitis: a pilot investigation from the Western Cape

**DOI:** 10.3389/froh.2025.1640915

**Published:** 2025-06-26

**Authors:** Salma Kabbashi, Yvonne Prince, Ndonwi Elvis Ngwa, Haly Holmes, Glenda Mary Davison, Saarah F. G. Davids, Manogari Chetty

**Affiliations:** ^1^ Department of Craniofacial Biology, Pathology, & Radiology, Faculty of Dentistry, University of the Western Cape, Cape Town, South Africa; ^2^ SAMRC/CPUT/Cardiometabolic Health Research Unit, Department of Biomedical Sciences, Faculty of Health & Wellness Sciences, Cape Peninsula University of Technology, Cape Town, South Africa; ^3^ Department of Oral Medicine & Periodontology, Faculty of Dentistry, University of the Western Cape, Cape Town, South Africa

**Keywords:** periodontitis, periodontal health, bacterial profile, 16S rRNA, *Fusobacterium*, diversity measures, South Africa

**Correction on**
Feasibility and preliminary findings of a bacterial diversity study in periodontitis: a pilot investigation from the Western Cape By Kabbashi S, Prince Y, Ngwa NE, Holmes H, Davids SFG and Chetty M (2025). Front. Oral Health. 6:1568393. doi: 10.3389/froh.2025.1568393

In the published article, there was an error in the author list, and author Glenda Mary Davison was erroneously excluded. The corrected author list appears below:

Salma Kabbashi1*, Yvonne Prince2, Ndonwi Elvis Ngwa2, Haly Holmes3, Glenda Mary Davison2, Saarah F. G. Davids2 and Manogari Chetty1

