# Supplementary material for: Feasibility and preliminary findings of a bacterial diversity study in periodontitis: a pilot investigation from the Western Cape
Source: Front Oral Health. 2025 Apr 23;6:1568393. doi: 10.3389/froh.2025.1568393 (PMC12055840; doi:10.3389/froh.2025.1568393)
Supplement: Supplementary file 1 [file Table1.docx]

Supplementary Material

**Supplementary Figure 1.** Box plots depicting statistical significance among top 10 genera between periodontitis cases vs. controls at genus level.


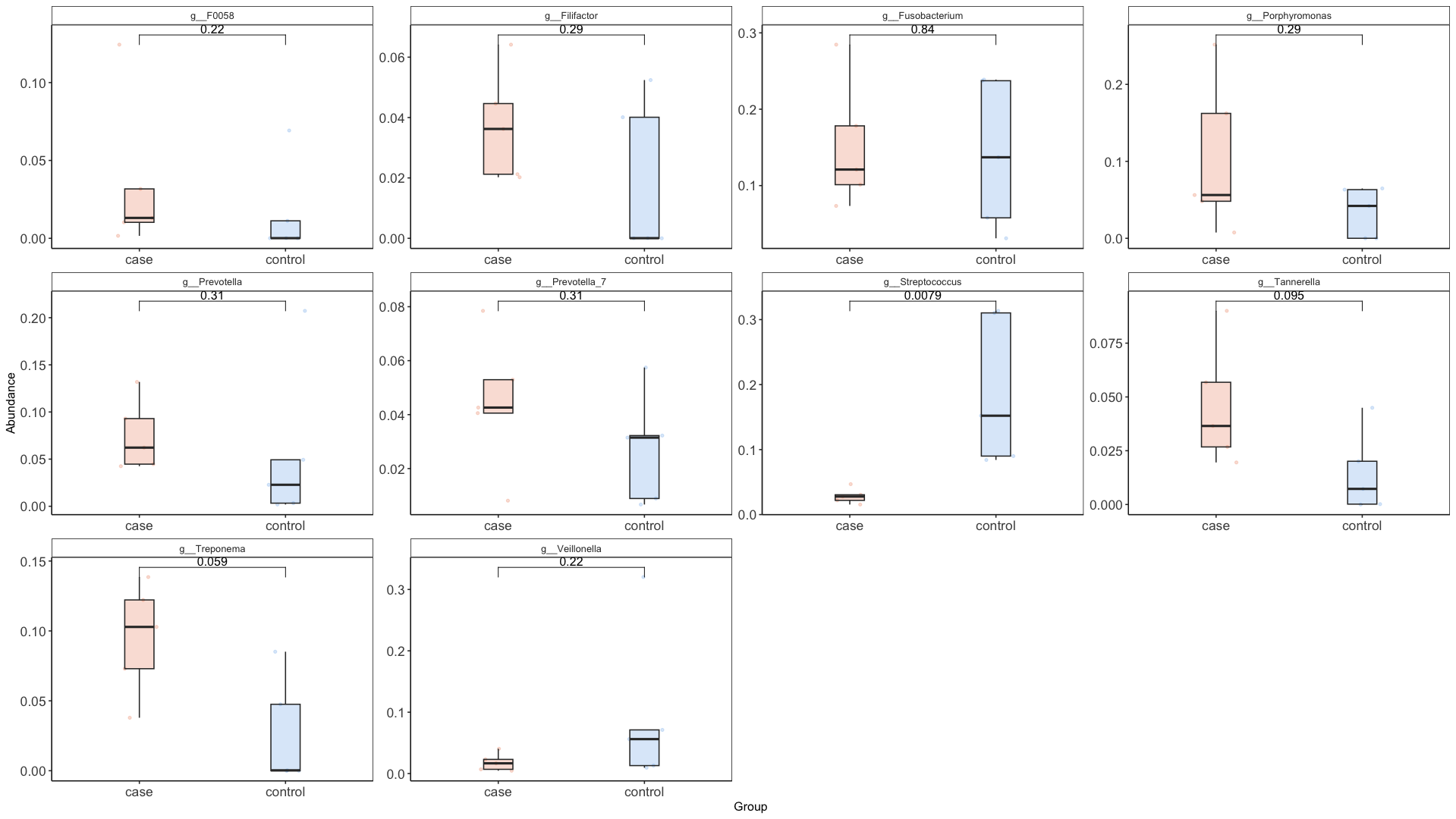


**Supplementary Figure 2.** Box plot depicting statistical significance among top 10 genera between grade B vs. C at genus level.


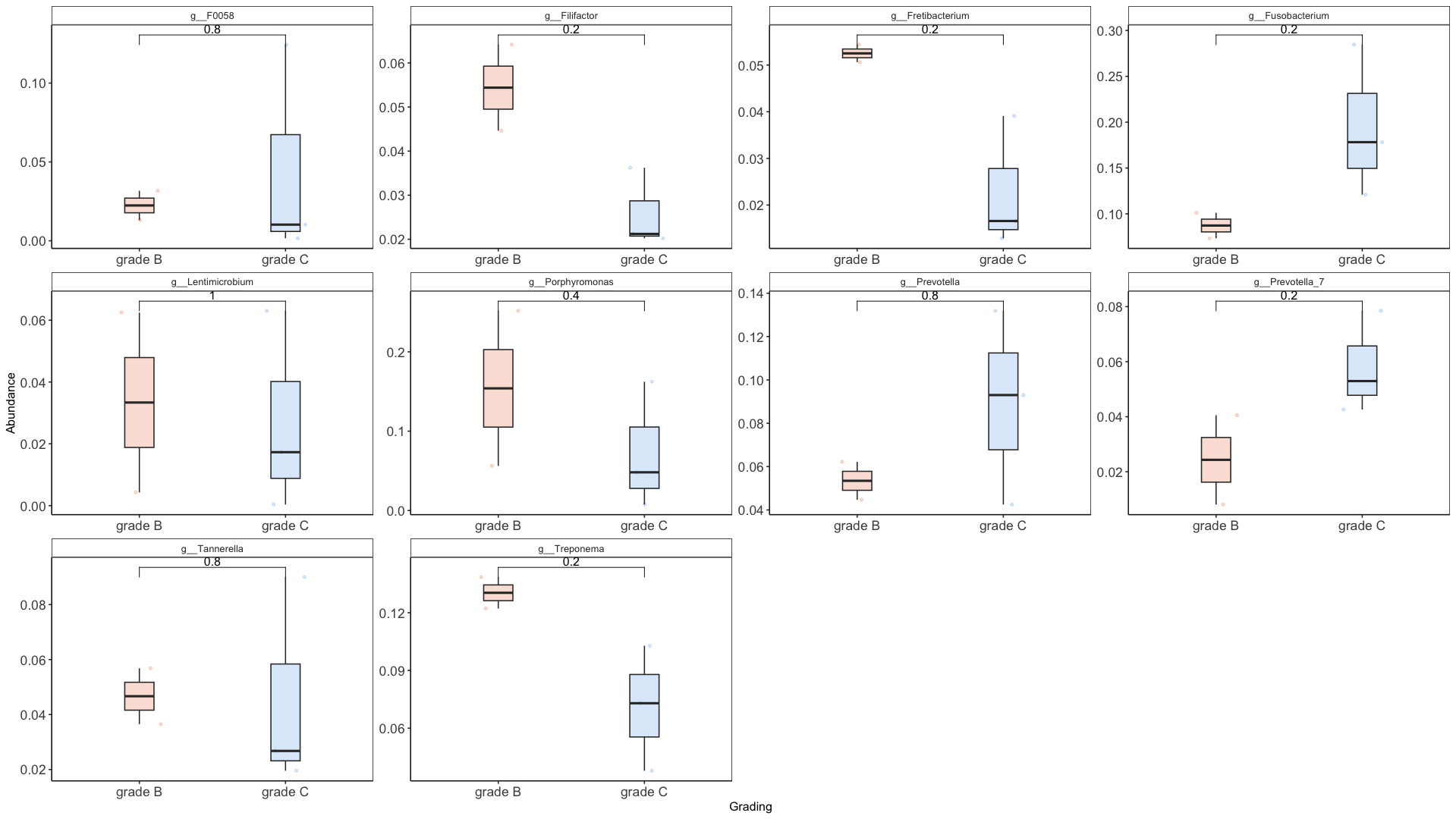


**Supplementary Figure 3.** Violin plots displaying the distribution of alpha-diversity measured by the ACE, Chao1, Observed, Shannon and Simpson indices for periodontitis cases and control groups.


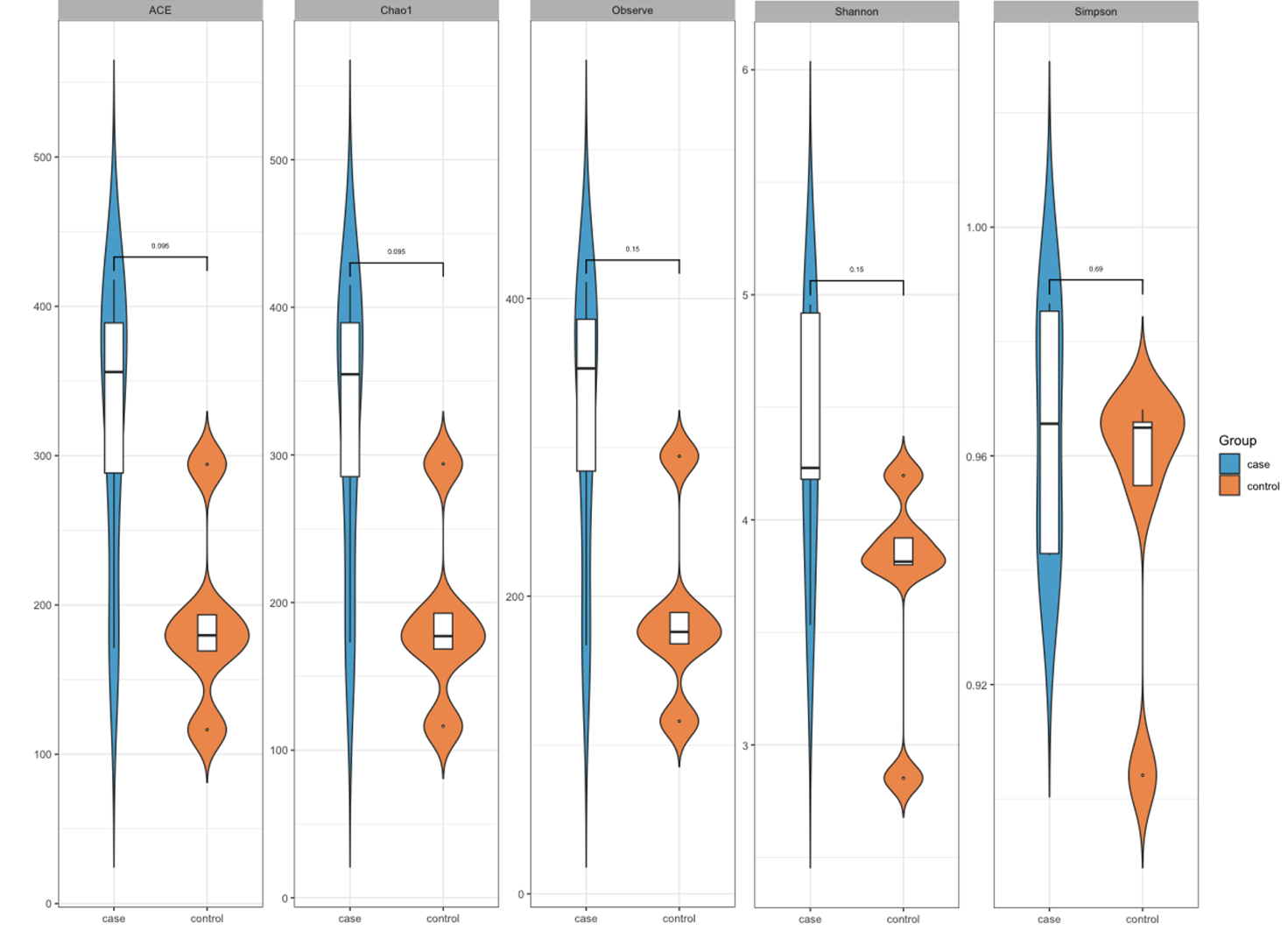


**Supplementary Table 4.** Values of multiple alpha-diversity and richness indices conducted for cases and controls samples.

| **Sample** | **Observed** | **Chao1** | **se.chao1** | **ACE** | **se.ACE** | **Shannon** | **Simpson** |
| --- | --- | --- | --- | --- | --- | --- | --- |
| Controls | | | | | | | |
| **1** | 115 | 115.5 | 1.026934056 | 115.8101084 | 5.259572599 | 2.852067628 | 0.9039755244 |
| **2** | 190 | 193.2727273 | 3.086955824 | 193.7084113 | 6.563628455 | 3.811034511 | 0.9647637373 |
| **3** | 175 | 176.3636364 | 1.736784992 | 178.6090099 | 5.363075366 | 3.920101107 | 0.9681608946 |
| **4** | 168 | 168.1666667 | 0.5434150007 | 168.6374131 | 4.996565429 | 3.801395513 | 0.9547883056 |
| **5** | 294 | 294 | 0.07130699079 | 294.1869895 | 6.807907581 | 4.194680708 | 0.9658344357 |
| Cases | | | | | | | |
| **1** | 410 | 417.1578947 | 4.746744186 | 419.3080924 | 8.30736868 | 4.914904171 | 0.98522035 |
| **2** | 388 | 391.2142857 | 2.916034795 | 392.1762721 | 7.637068785 | 4.955471951 | 0.9865854495 |
| **4** | 167 | 168 | 1.449748541 | 169.0088136 | 5.508885157 | 3.537625937 | 0.9427968112 |
| **4** | 352 | 355.9285714 | 3.337433017 | 356.4922415 | 8.164123841 | 4.198579067 | 0.9435529747 |
| **7** | 283 | 288.037037 | 3.505684016 | 293.6519906 | 7.701109048 | 4.227267085 | 0.9654163371 |

Se.: standard error; ACE: Abundance Coverage Estimator.
